# Development of a self-management support practice framework for addressing cancer-related fatigue: a modified Delphi study

**DOI:** 10.1007/s11764-023-01348-7

**Published:** 2023-02-24

**Authors:** Oluwaseyifunmi Andi Agbejule, Nicolas H. Hart, Stuart Ekberg, Raymond Javan Chan

**Affiliations:** 1 Caring Futures Institute, College of Nursing and Allied Health, Flinders University, Bedford Park, South Australia 5042 Australia; 2 Cancer and Palliative Care Outcomes Centre, Queensland University of Technology, Kelvin Grove, Queensland 4059 Australia; 3 Centre for IMPACCT, Faculty of Health, University of Technology, NSW Sydney, 2007 Australia; 4 School of Psychology & Counselling, Faculty of Health, Queensland University of Technology, Kelvin Grove, Queensland 4059 Australia

**Keywords:** Cancer survivors, Fatigue, Implementation, Survivorship, Self-management, Taxonomy

## Abstract

**Purpose:**

Managing cancer-related fatigue requires individuals to adopt a range of self-management behaviours. However, clinicians report the lack of clear guidance on self-management support practices hinders their provision of supportive care. To develop consensus on a framework of core practices required by health professionals to deliver effective self-management support to cancer patients and survivors experiencing cancer-related fatigue.

**Methods:**

A preliminary framework of 47 practice items (14 Key Practices, 33 Practice Components) was derived from a systematic review, and a self-management support capability outline for primary care professionals. This preliminary framework was presented for consensus rating and comment in a two-round modified Delphi study conducted with a panel of health professionals, research academics, and cancer consumers.

**Results:**

Fifty-two panel participants comprising consumers (*n* = 25), health professionals (*n* = 19), and researchers (*n* = 16) were included in Round 1 of the modified Delphi study. Feedback from the panel produced consensus on retaining 27 of 47 original practice items without change. Seventeen items (including 12 modified, and 5 newly created practice items) were sent to the panel for rating in Round 2. Thirty-six experts produced consensus on retaining all 17 practice items in Round 2. The final framework comprised 44 items (13 Key Practices, 31 Practice Components).

**Conclusions:**

The practice framework offers an evidence- and consensus-based model of best practice for health professionals providing self-management support for cancer-related fatigue.

**Implications for Cancer Survivors:**

This framework is the first to focus on quality provision of self-management support in managing cancer-related fatigue, one of the most prevalent symptoms experienced by cancer patients and survivors.

**Supplementary information:**

The online version contains supplementary material available at 10.1007/s11764-023-01348-7.

## Introduction

Cancer-related fatigue is one of the most common and distressing symptoms reported by people affected by cancer (i.e. cancer patients and survivors) [1]. This fatigue is commonly defined as a distressing persistent subjective sense of physical, emotional, or cognitive exhaustion related to cancer and cancer treatment that is not proportional to recent activity and interferes with usual functioning [2]. Reported detrimental effects on quality of life include substantial reductions in physical, mental, emotional, and social wellbeing [3, 4], with evidence also indicating that associated adverse effects can persist long after treatment cessation [3, 5–7].

Management of cancer-related fatigue involves the adoption of self-management behaviours (e.g. increasing physical activity and exercise; using energy conservation strategies; employing mind–body practices) [8–14]. Engagement in fatigue self-management behaviours can be complex and can often require patients and cancer survivors to recognise, track, self-monitor, self-report, and apply problem-solving skills to manage their fatigue along with their other comorbid conditions [15]. People affected by cancer therefore require access to holistic self-management support.

Effective management strategies for cancer-related fatigue are well established with the existence of a plethora of clinical guidelines including the National Comprehensive Cancer Network (NCCN) Guidelines for Cancer-Related Fatigue [2], the Canadian Association of Psychosocial Oncology (CAPO) Pan Canadian Guidelines for Cancer-related Fatigue [16], and the European Society for Medical Oncology (ESMO) Clinical Practice Cancer-related Fatigue Guidelines [17]. Although these clinical guidelines advise health professionals on what strategies they ‘should’ recommend to those experiencing cancer-related fatigue (e.g. *advise patients to engage in moderate intensity of physical activity 30 min per day, 5 days per week as tolerated* [16]); they do not provide guidance on ‘how’ health professionals can support individuals to undergo behavioural change and *adopt* these management strategies (e.g. by providing tools to assist with exercise, creating goals and actions plans that are regularly reviewed, etc.).

Health professionals regularly report they frequently lack the confidence, knowledge, and ability to provide effective self-management support to those experiencing cancer-related fatigue [18–21]. Clinicians often describe existing clinical guidance as lacking the relevant information for facilitating self-management support [18, 19, 22]. There is a need for specific guidance for health professionals to facilitate evidence-based fatigue self-management strategies [18, 22–24]. The aim of the current study is to establish a *best practice framework* for facilitating effective self-management support for cancer-related fatigue.

## Methods

This study involved international experts in fatigue, self-management, and supportive care to focus on developing guidance on how health professionals can support cancer survivors to optimise self-management of their fatigue. A modified Delphi study design was used to generate expert consensus on the core practices required by health professionals to deliver effective self-management support to people affected by cancer (i.e. cancer patients and survivors) experiencing cancer-related fatigue. These core practices were then included in a practice framework (defined below). In this study, we adopted a structured group consensus strategy that systematically uses high-quality literature, the opinion of stakeholders, and judgement of industry specialists to reach an agreement and determine content validity [25]. We used an anonymous and iterative feedback process to establish consensus on a specific topic of interest with an invited panel of ‘experts’ over a series of rounds.

### Defining a practice framework

Practice frameworks have been defined and used variably by different professions [26, 27]. This study is informed by the definition used by Connolly [26] and Stanley and colleagues [27], where a ‘practice framework’ is summarised as a “template not based on or informed by organisational imperatives (e.g. budgets or compliance edict) but designed through and informed by value-based practice research, and evidence; that offers a mapping out of what we do and why, offering a rationale for practice, while promoting a range of practice tools for assessments and intervention”. In the context of the current study, ‘value-based practice’ refers to an approach to supporting clinical decision-making, which provides practical skills and tools for eliciting individual values and negotiating these with respect to best available evidence [28]. More specifically, our proposed framework focuses on the health professional, and presents the ‘practices’ or tasks required to provide holistic self-management support for people affected by cancer experiencing acute and long-term cancer-related fatigue. The proposed practice framework also aims to reinforce what constitutes ‘best’ practice for cancer-related fatigue self-management support.

### Developing the preliminary framework

**Fig. 1:**
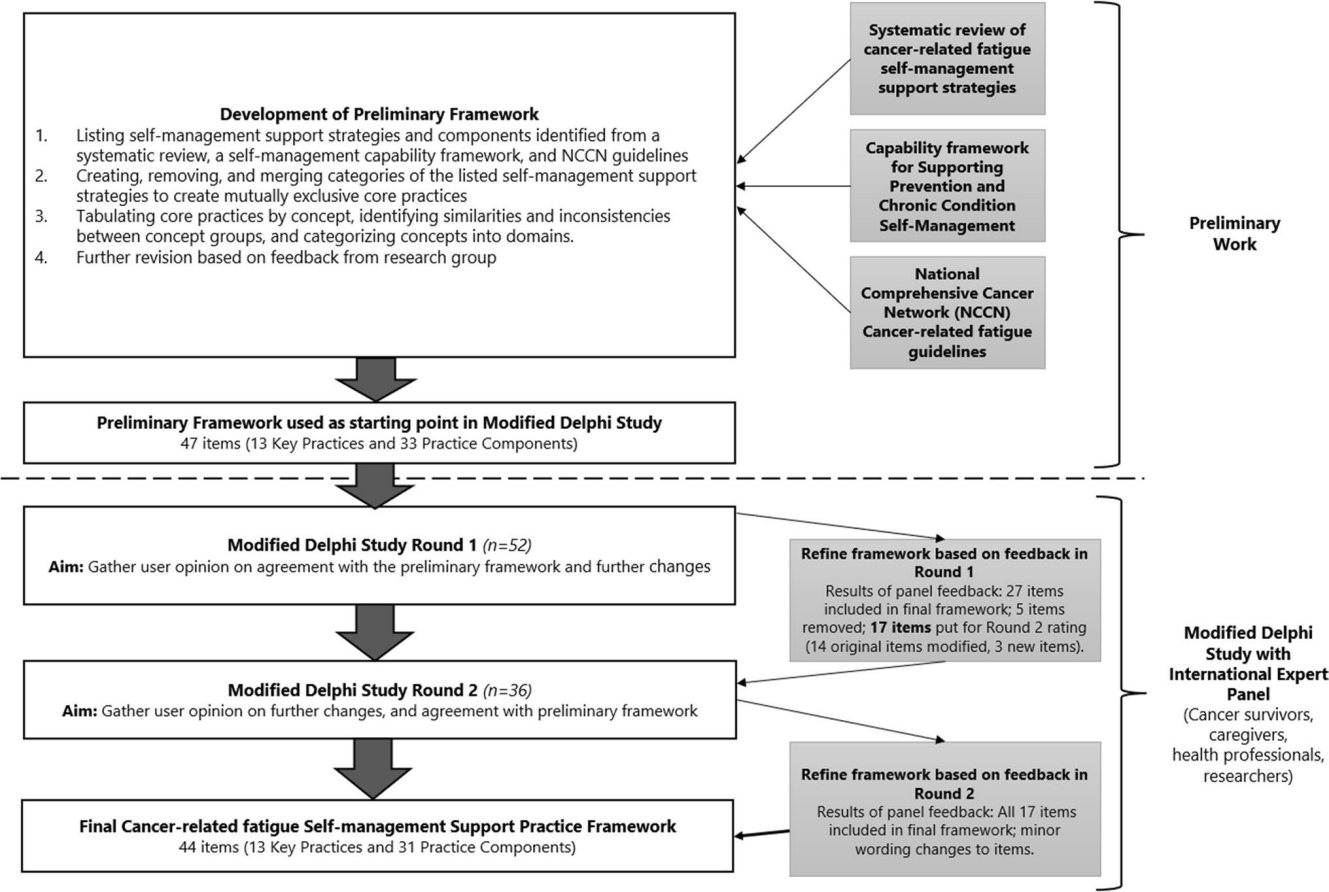
Delphi study process

An initial, preliminary framework was developed by the research team (OAA, NHH, SE, RJC). The preliminary framework was created through a qualitative synthesis of a formative systematic review of self-management programmes for cancer-related fatigue conducted by the research team [29], and a self-management support capability framework for primary care professionals [30] (see Fig. 1). Cancer-related fatigue management strategies from the National Comprehensive Cancer Network (NCCN) Fatigue Guidelines [2] were also incorporated. Methods and results of the systematic review are described elsewhere [29]. Briefly, the systematic review examined 51 randomised controlled trials and highlighted the components of self-management support programmes that were effective. The primary care professional capability framework [30]presented the essential knowledge, attitudes, and skills needed by health professionals to support cancer patients and survivors to self-manage.

An iterative process was used to classify self-management support components, strategies, and capabilities identified through research [29]. This process involved creating, removing, and merging categories to create mutually exclusive ‘key practices’ and ‘practice components’. This was initially completed by one member of the research team (OAA) with the remaining members providing input, feedback, and suggesting changes (RJC, NHH, SE). Feedback was then incorporated by OAA. This iterative process was repeated until all members of the research team agreed on the structure and content. Key practices describe the proposed activities health professionals are required to undertake to provide best practice self-management support for cancer-related fatigue. Practice components describe the steps needed to complete a key practice. The core practices (comprising key practices and practice components) were then tabulated by concept, analysed for similarities and inconsistencies between concept groups, and then categorised into domains by all members of the research team (OAA, RJC, NHH, SE). The resulting preliminary framework consisted of 47 practice items (14 Key Practices and 33 Practice Components) categorised under five domains: (1) Establishing Context and Defining the Problem; (2) Developing an Action Plan; (3) Improving Patient Knowledge; (4) Training Rehearsal (Strategy Building); and (5) Care Co-ordination and Maintenance (Online Resource 1).

Support practices described in the framework were written to be applicable to the management of cancer-related fatigue at all stages (e.g. fatigue at diagnosis, treatment, immediately post-treatment and at later stages post-treatment), as practices and specific strategies were to be delivered based on an individual’s perceived level of fatigue severity and interference, regardless of cancer diagnosis, and treatment type, or phase in the cancer continuum.

### Participant recruitment and panel selection

Potential panel participants were identified through networks within the Multinational Association for Supportive Care in Cancer (MASCC)—including the fatigue and self-management subgroups; cancer consumer networks including Cancer Voices Australia, Canadian Cancer Survivor Network, and the Guyana Cancer Foundation; and other relevant groups or individuals identified by the research team. Potential participants received an invitation email providing study information and seeking expression of interest. To reduce participant attrition, potential participants were informed of expected time commitments and were asked to confirm their interest to participate in all modified Delphi rounds [31]. Criteria for inclusion into the modified Delphi panel included (1) being a minimum of 18 years of age; (2) having experience or involvement with cancer or cancer survivorship; and (3) being able to read and understand English to a proficient level. Potential panel participants were encouraged to distribute the expression of interest form to peers and colleagues, approximating a snowball sampling technique. Although there is no standard sample size for a Delphi panel, studies suggest a minimum of eight participants, with more members increasing the reliability and representativeness of group judgement [32]. We aimed to recruit a minimum of 40 participants for the first round of the modified Delphi study. This sample size takes into consideration the 50% attrition rate commonly reported in [31, 33], thus maximising the likelihood of a sufficient sample size in subsequent rounds of the study. A mailing list of 92 individuals was created through the email addresses obtained through the completed expression of interest forms and was used to distribute the practice items and surveys during the modified Delphi rounds. Surveys were created using Qualtrics XM (Qualtrics, Provo, UT) survey software and were pilot tested by the research team and other invited parties (i.e. cancer survivorship researchers, laypersons in the community) before being distributed to panel participants.

#### Round 1

The preliminary framework was emailed to panel participants through an online survey, accompanied by a consent form, an explanation of study objectives and the consensus process, and instructions. Panel participants were given the option to withdraw consent or refuse participation at any time. Each panel participant was asked to rate their agreement with each practice statement on a 5-point Likert scale (from ‘strongly disagree’ to ‘strongly agree’), indicating the degree that each practice statement should be considered best practice and included in the final practice framework. Panel participants were also given the option to provide comments and suggest additional items that may not have been included when initially developing practice items. Survey responses were then qualitatively and quantitatively analysed to appraise consensus among participants.

#### Round 2

Practice items that did not reach consensus in Round 1, or were newly created or modified based on Round 1 panel feedback, were sent back to panel participants for another round of voting. Individuals who did not participate in Round 1 were also invited to participate in Round 2, as evidence demonstrates this leads to better representation of originally invited panel participants’ opinions, reduces the chance of ‘false consensus’, and does not compromise the outcome of the Delphi process [34]. Participants were provided with a document summarising the changes made from participant feedback in Round 1. Items that did not meet consensus in Round 2 were not included in the final framework.

#### Analysis

Survey responses were imported into the statistical software programme Jamovi (Version 2.3) for analysis. Descriptive statistics such as frequency, mean, median, percentage, and range were used to describe demographic data. Statistics such as the median and interquartile range (IQR) were derived and evaluated for each practice item [35]. Consensus on participant opinions was defined as IQR ≤ 1—a highly recommended rigorous and objective; widely accepted; and frequently used threshold for Delphi studies [25, 35–37]. As per recommendations in the literature, median scores were used to summarise participant agreement with a statement [37]. A group median of 4–5 was considered to indicate agreement, and 1–2 disagreement. The percentage of panel participants responding to a given category was also recorded. Free-text responses were examined using basic thematic analysis methods [38]. Thematic analysis focused on concepts and categories used by participants in their free-text responses. Analysis iteratively progressed from identifying specific ideas to conceptualising high-level explanations that constitute a patterned response, or ‘theme’. These themes were then used to inform changes to the practice framework.

## Results

### Demographics

#### Round 1

Of the 92 individuals invited to participate, 52 completed the Round 1 survey (56.5% response rate). Demographic characteristics of participants in each round are presented in Table 1.

**Table 1:** Demographics of panel participants

	Round 1(*n* = 52)	Round 2(*n* = 36)
	*N*, %	*N*, %
Sex		
Female	37 (71.2)	26 (72.2)
Male	16 (30.8)	10 (27.8)
Did not answer	1 (1.9)	
Age group		
18–35 years	5 (9.6)	3 (8.3)
36–45 years	9 (17.3)	6 (16.7)
46–55 years	8(15.4)	5 (13.9)
56–65 years	15 (28.8)	11 (30.6)
Over 65 years	15 (28.8)	11 (30.6)
Race		
Asian	4 (7.7)	3 (8.3)
Multiracial or biracial	1 (1.9)	
Black, African, or African American	1 (1.9)	
White or Caucasian	37 (71.2)	23 (63.9)
Hispanic or Latino		1 (2.8)
A race/ethnicity not listed here	9 (17.3)	9 (25.0)
Participant type		
Cancer survivor	25 (48.1)	16 (44.4)
Clinician	19 (36.5)	13 (36.1)
Researcher	16 (30.8)	11 (30.6)
Family/caregiver	1 (1.9)	3 (8.3)

Clinician/research academic data	(*n* = 28)	(*n* = 21)
Occupation field		
Medicine	14 (50.0)	8 (38.1)
Nursing	6 (21.4)	6 (28.6)
Allied health	3 (10.7)	2 (9.5)
Psychology	2 (7.1)	
Primary care	1 (3.6)	1 (4.8)
Social work	1 (3.6)	1 (4.8)
Epidemiology	1 (3.6)	
Public health		1 (4.8)
Researcher/advisor		1 (4.8)
Physical medicine and rehabilitation		1 (4.8)
Region of work		
Europe	14 (50.0)	9 (42.9)
North America	8 (28.6)	6 (28.6)
Oceania	4 (14.3)	5 (23.8)
Asia	2 (7.1)	1 (4.8)
Years of experience in cancer care (research or clinical care)	Ranged from 2 to 45 years, with a median of 15 years	Ranged from 1 to 35 years, with a median of 14 years

Cancer survivor and family/caregiver data	(*n* = 25)	(*n* = 17)
Primary cancer site of cancer survivor		
Solid tumours	20 (80.0)	12 (70.6)
Haematological malignancies	5 (20.0)	4 (23.5)
Did not answer		1 (5.9)
Years since cancer diagnosis		
1–2 years	1 (4.0)	1 (5.9)
2–5 years	2 (8.0)	2 (11.8)
5–10 years	9 (36.0)	5 (29.4)
More than 10 years	13 (52.0)	8 (47.1)
Did not answer		1 (5.9)
Region of residence		
Oceania	16 (64.0)	11 (64.7)
North America	7 (28.0)	3 (17.6)
Europe	2 (8.0)	2 (11.8)
South America		1 (5.9)

Most participants identified as White or Caucasian (*n* = 37/52; 71.2%); female (*n* = 37/52; 71.2%); and indicated they were aged between the age groups 56–65 (*n* = 15/52; 28.8%) or over 65 years (*n* = 15/52; 28.8%). Twenty-four of 52 (46.2%) panel participants indicated their participant type as solely a cancer patient or survivor, 11 (21.2%) indicated they were health professionals only, and 9 (17.3%) panel participants identified solely as researchers or academics. The remaining participants selected more than one participant type with seven (13.5%) indicating they were health professionals and researchers, and one recording they were a health professional, cancer patient or survivor, and caregiver.

Out of the 28 research academics and health professionals, most indicated their occupation was in the field of medicine (*n* = 14/28; 50%), followed by nursing (*n* = 6/28; 21.4%), with the remainder in various allied health roles. Years of experience in research or clinical cancer care ranged from 2 to 45 years, with a median of 15 years of experience. Region of residence varied across Europe (*n* = 14/28; 50%), North America (*n* = 8/28; 28.6%), Oceania (*n* = 4/28, 14.3%), and Asia (*n* = 2/28; 7.1%). Out of the 25 cancer consumers (cancer patients or survivors; and family members or caregivers), most resided in Australia (*n* = 16/25; 64%); indicated breast as the primary cancer site (*n* = 9/25; 36%); and indicated it had been more than 10 years since first cancer diagnosis (*n* = 13/25; 52%).

#### Round 2

Of the 92 invited individuals, 36 completed the Round 2 survey, resulting in a 39.1% response rate. Most participants identified as White or Caucasian (*n* = 23/36; 63.8%), female (*n* = 25/36; 69.4%); and indicated they were aged between the age groups 56–65 (*n* = 11/36; 32.4%) or over 65 years (*n* = 11/36; 32.4%). Twenty-one (*n* = 21/36; 58.3%) participants indicated they were health professionals or research academics and 17 (*n* = 17/36; 47%) were cancer patients and survivors or family and caregivers. Further demographic characteristics of Round 2 participants can be found in Table 1.

### Consensus building

#### Round 1

Quantitative consensus (IQR ≤ 1, and median of 4 to 5) on whether a key practice or practice component was to be included in the final framework was achieved for all practice items in Round 1. Themes identified from the panel participants’ free-text responses were also considered by the research team to ensure a co-creative process with participants, whereby the modification and inclusion of practice items were consistent with panel feedback.

In addition to feedback on grammar and formatting changes, priority areas for framework revisions from the panel’s written feedback were as follows: (1) the need to consider patient preferences for peer and familial support; (2) considering the needs of the patient’s support network; (3) the importance of tailoring support information to different learning needs; (4) identifying risk factors for cancer-related fatigue; and (5) emphasis on referral to other health professionals for continual care. Identified themes and subsequent responses or amendments by the research team are presented in Online Resource 2.

As a result of panel feedback, 27 practice items from Round 1 were designated for inclusion in the final practice framework (Fig. 1). Fourteen practice items were modified, and three new items were added and were included in Round 2 for rating. Five items were removed. Specific changes to practice items based on Round 1 panel feedback are displayed in Online Resource 1. Finally, in addition to the practice items, consensus was reached on the definition and components of a cancer-related fatigue self-management support action plan (Online Resource 3).

#### Round 2

All practice items submitted for feedback in Round 2 (*n* = 17) reached consensus for inclusion into the final framework. Qualitative comments included suggestions for wording changes. The research team discussed the minor wording proposals from the panel and accepted or rejected them before finalising the framework. These changes did not go back to panel members for endorsement through a third round, as they were not of sufficient scope for reappraisal (e.g. changes to grammar, spelling). Some panel participants also suggested that greater clarity and specificity was required for the practice components to be useful for implementation, and teaching and evaluating practice. To address this, a decision was made to re-incorporate the accompanying examples and contextual elements that were included in Round 1. These were moved back into the main practice framework (and not just as supplemental material—see Online Resource 2 for further detail). As a result of panel feedback from Round 2, all items were included in the final framework. Thus, the final practice framework consisted of 13 Key Practices and 31 Practice Components. Table 2 depicts the domains and key practices of the framework; the full practice framework is presented in Online Resource 3.

**Table 2:** Domains and key practices of self-management support practice framework

Establishing Context and Defining the Problem
1. Collect and use clinical and behavioural information to inform decision-making about the patient’s self-management of cancer-related fatigue 2. Assess the patient’s capacity for self-management
Developing an action plan
3. Create a cancer-related fatigue management action plan in collaboration with the patient that incorporates evidence-based coping strategies that are aligned with patient preferences
Improving patient knowledge
4. Provide tailored evidence-based information on cancer-related fatigue and common management strategies in a diversity of formats to accommodate different learning styles 5. Provide tailored evidence-based information on managing common psychological consequences of cancer and cancer-related fatigue in a variety of formats to accommodate different learning styles 6. Provide tailored evidence-based information about available social support in a variety of formats to accommodate different learning styles and check patient understanding
Training rehearsal (strategy building)
7. Provide the patient with problem-solving and evidence-based solution-focused strategies to communicate with their systems of support (includes health professionals, non-health professionals, personal communities; and voluntary and community groups) about cancer-related fatigue 8. Provide evidence-based coaching for lifestyle modifications that support living with cancer-related fatigue 9. Provide the patient with evidence-based problem-solving strategies for coping with the psychological effects or risk factors of cancer-related fatigue 10. Provide evidence-based health promotion and education on lifestyle adaptation strategies 11. Provide regular review of self-management activities, and self-management goals and action plans in collaboration with the patient, their support network (with the patient’s consent), and their health care team
Care co-ordination and Maintenance
12. Provide practical support that facilitates ongoing self-management 13. Attend to requests to review the symptoms of cancer-related fatigue

## Discussion

To our knowledge, this study is the first to develop a framework of core practices required by health professionals to deliver effective self-management support to people affected by cancer (i.e. cancer patients and survivors) experiencing cancer-related fatigue. This framework had input from an international panel of cancer consumers, health professionals, and cancer researchers. The modified Delphi study that was used established resounding consensus on the best clinician practices for facilitating cancer-related fatigue self-management support at all phases of the cancer continuum.

For self-management to be effective, cancer patients and survivors must be supported in managing their symptoms and conditions. Health professional guidance for fatigue management often lacks detail about effective self-management support, contributing to the provision of inadequate and limited support [18, 19, 21, 29] (e.g. information provision alone, normalisation of fatigue symptoms, advising individuals to simply rest and relax).

The practice framework presented in this study considers the complex nature of cancer-related fatigue management, by conceptualising self-management as an active process that requires an essential set of collaborative-and partnership building behaviours, skills, knowledge, and attitudes. Further, it presents the ideal practices needed to effectively facilitate the adoption of fatigue self-management behaviours. These include action planning, motivational interviewing, and assessment of self-management capacity to facilitate self-management through enhanced self-efficacy. This framework could be used by clinicians as a tool to guide their provision of cancer-related fatigue self-management support. This framework may also allow clinicians to evaluate current practice, determine professional development needs, and support their understanding of the holistic nature of effective cancer-related fatigue self-management support.

Although primarily directed to health professionals, this practice framework may have functionality across several settings (see Box 1). Clinical leaders and educators could use the framework to build awareness and knowledge among their clinical teams. Researchers could use the framework to synthesise evidence on cancer-related fatigue self-management Consumers could refer to the framework to advocate for their care, and develop their own understanding on the various roles of self-management support for cancer-related fatigue.

Box 1 Use of the cancer-related fatigue self-management support practice framework.

**Table:** 

The following recommendations are designed to help individuals and organisations make the best possible use of the Practice Framework
For the individual health professional
• Use the Practice Framework as a tool:
○ to guide the provision of self-management support for cancer-related fatigue
○ for determining your professional development needs
○ for evaluating current practice when providing support to those managing cancer-related fatigue
• Use the Practice Framework and associated learning resources to undertake self-directed learning
• Refer colleagues new to working with people affected by cancer (i.e. cancer survivors, cancer patients) and/or cancer-related fatigue to the framework
• Use the Practice Framework to develop an understanding about:
○ the extent of cancer-related fatigue impact on those affected by cancer and the importance of its management
○ the various roles of different health professionals in the delivery of self-management support for cancer-related fatigue
For the clinical leader/ educator
• Use the Practice Framework as a tool to:
○ develop clinician awareness and knowledge of evidence-based cancer-related management and assessment strategies
○ provide training/in-service programmes to improve ability to undertake practices (how to use certain questionnaires, practice developing an action plan, etc.)
○ advocate for system-level changes to provide resources (time, space, and human) to deliver optimal support for cancer-related fatigue management
For the cancer-related fatigue self-management intervention/programme developer
• Use the Practice Framework to aid development of a cancer-related fatigue self-management intervention/programme (determining the specific components that are needed)
For the researcher
• Use the Practice Framework as a tool to:
○ describe self-management support interventions for cancer-related fatigue
○ synthesise evidence on cancer-related fatigue self-management
For the consumer experiencing cancer-related fatigue (and their family/carer’s)
• Use the Practice Framework as a tool to:
○ develop understanding of the various roles of different health professionals in the delivery of self-management support for cancer-related fatigue
○ Advocate for improved delivery of cancer-related fatigue management support
○ advise your health care team(s) about the existence of the practice framework and teaching and learning resources in efforts to improve your care

Although panel participants acknowledged framework components as best practice, comments identified a need for further clarity around healthcare professional responsibility (i.e. who should do what). Cancer-related fatigue is multifactorial [1, 39], meaning that there are diverse factors that can contribute to, or cause it (e.g. cancer type, treatment type, anaemia, nutrition factors, psychological factors, etc.). It is therefore expected that the provision of cancer-related fatigue self-management support will require a multidisciplinary approach. If a multidisciplinary approach is adopted, it will not be necessary for all healthcare professionals to be proficient and have the commitment to deliver all practice components, especially when these fall outside of a professional’s expertise or scope of practice. However, this practice framework does allow for healthcare professionals to identify areas of care provision that may be achievable within their clinical care domain, and where additional training or collaboration may be encouraged or required.

The execution of the key practices and practice components specified in the framework may require health professionals to apply, adapt, and integrate new and existing evidence-based knowledge or seek professional development opportunities. This framework does not provide, present, or describe the capabilities or competencies required by health professionals, as these are already described in clinical practice guidelines [1, 2, 10, 24, 25]. Rather, the practice framework outlines the support tasks that health professionals and health care teams should undertake when supporting people affected by cancer to self-manage their fatigue.

The consumers involved in this study emphasised the importance of health professionals not merely providing information on self-managing fatigue, but delivering information in a way that promotes the understanding and knowledge of the consumer. Although not explicitly listed in each practice item, we stress that the execution of practices outlined in the framework should be underpinned by the presence of effective, person-centred, health professional communication which involves the ability to establish and develop mutual understanding, rapport, trust, respect, and cooperation with people affected by cancer using clear and plain language. This includes making appropriate adjustments (e.g. use of appropriate language and detail, use of appropriate verbal and non-verbal cues, confirming that the other person has understood) to meet the communication and information needs of patients and their support network (e.g. caregivers, family, friends) and providing opportunities for the patient and their support network to demonstrate their understanding.

### Future work

This study has identified the requisite practices needed to effectively deliver fatigue self-management support. Although some feedback to enhance framework usability and implementation was received and incorporated, future work could involve further consultation with key stakeholders. This consultation could be used to enhance understanding of stakeholders’ perspectives about the acceptability and relevance of the framework to specific clinical, educational, and cultural contexts, and among underserved or high-risk groups. Future work could also identify different stakeholders’ needs in supporting the implementation of the framework in their local setting. This includes fine-tuning the language and presentation of the framework for different contexts (e.g. ‘cheat sheets’, communication tools, role play scenarios for training, flow diagrams, etc.) and determining educational and training requirements. Stakeholder consultation could also be used to further define the roles of different professional disciplines in providing self-management support for cancer-related fatigue. Such developments would fine-tune the framework to provide clinical and implementation guidance that encourages clear professional judgement and explicit decision-making.

### Strengths and limitations

Strengths of this study include its online anonymous nature, which allowed for unrestricted expression of panel opinions. This helped reduce the influence of dominant personalities and the effect of panellists’ status on results [37]. However, the online forum limited the opportunity for robust discussion. Another strength of our work is that the modified Delphi study comprised representation from a diverse international panel of consumers, health professionals, and cancer researchers from varying continents, professional fields, and clinical settings. Although the resulting practice framework incorporated diverse international perspectives, the panel was not representative of participants from every country/region, culture, setting, or scope of practice. Cultural influences on health, fatigue, compliance, and attitudes towards care will need to be considered when adapting the framework to different contexts [40]. Limiting panel eligibility criteria to individuals proficient in English could have resulted in potential candidates and viewpoints being missed.

## Conclusion

This modified Delphi study presents a framework for health professionals that outlines the essential support practices needed to facilitate the uptake of cancer-related fatigue management strategies. Future work is needed to assess the clinical utility and implementation (including evaluation of such implementation) of the practice framework. The provision of holistic self-management support by health care teams is key for the uptake and integration of evidenced-based fatigue management strategies into clinical practice and for improving the outcomes of patients and cancer survivors.

### Supplementary information

Below is the link to the electronic supplementary material.

Supplementary file1 (DOCX 37.8 KB)

Supplementary file2 (PDF 397 KB)

## Data Availability

The datasets generated during and/or analysed during the current study are available from the corresponding author on reasonable request.
