# Supplementary material for: Development of a self-management support practice framework for addressing cancer-related fatigue: a modified Delphi study
Source: J Cancer Surviv. 2023 Feb 24;18(3):972–82. doi: 10.1007/s11764-023-01348-7 (PMC11082027; doi:10.1007/s11764-023-01348-7)
Supplement: Supplementary file 1 — Supplementary file1 (DOCX 37.8 KB) [file 11764_2023_1348_MOESM1_ESM.docx]

**Online Resource 1:** Changes from Round 1

| Original Practice Statement | Modified Statement based on Panel Feedback |
| --- | --- |
| **DOMAIN 1: ESTABLISHING CONTEXT AND DEFINING THE PROBLEM** | |
| **Key Practice 1** | |
| 1. Collect and use clinical and behavioural information to inform decision making about the patient's self-management of cancer-related fatigue | Reached consensus for inclusion in Round 1 and included in final framework |
| 1a) Conduct a consultation and assessment with the patient, and if available, other key people in the patient’s support network (e.g., carer’s; family members) to collaboratively define key concerns, problem areas and priorities. | Reached consensus for inclusion in Round 1 and included in final framework |
| **NEW** | 1b) Identify family members/informal caregivers need to have guidance from healthcare providers to facilitate their ability to support the patient with self-management strategies. |
| 1c) Collect, record, and store key clinical, symptom and behavioural information. | 1c) Collect and record key clinical, symptom, behavioural, **and psychological information and highlight risk factors that may contribute to the patient’s cancer-related fatigue.** |
| **NEW** | 1e) Conduct a fatigue (e.g., Brief Fatigue Inventory Questionnaire, informal questioning) to determine fatigue severity, onset, duration, pattern, associated patient distress, and interference with daily living. |
| **Key Practice 2** | |
| 2. Assess the patient's capacity for self-management. | Reached consensus for inclusion in Round 1 and included in final framework |
| 2a) Identify factors within the patient, and the patient's physical and social environment, that they perceive may improve or worsen their cancer-related fatigue (e.g. more severe fatigue in the afternoon). | Reached consensus for inclusion in Round 1 and included in final framework |
| 2b) Identify the patient's beliefs, attitudes, and knowledge about cancer-related fatigue, and identify their current coping strategies. | 2b) Identify the patient's **and the informal caregivers/ support network** beliefs, attitudes, and knowledge about cancer-related fatigue, and identify their current coping strategies. |
| 2c) Identify factors (i.e., cultural consideration, language literacy levels, availability of peer support network, pre-existing conditions) that may affect the patient's ability to participate in self-management activities. | Reached consensus for inclusion in Round 1 and included in final framework |
| **DOMAIN 2: DEVELOPING AN ACTION PLAN** | |
| **Key Practice 3** |  |
| 3. Create a cancer-related fatigue management action plan in collaboration with the patient | Create a cancer-related fatigue management action plan in collaboration with the patient **that incorporates evidence-based coping strategies that are aligned with patient preferences.** |
| 3a) Reflect on the patient’s capacity for self-management (including barriers), and the clinical and behavioural information gathered during pre-assessments, to refine the patient’s priorities, needs, and goals concerning their cancer-related fatigue and general lifestyle. | Reached consensus for inclusion in Round 1 and included in final framework |
| 3b) Plan a culturally and contextually relevant fatigue self-management care plan drawing on clinical and psychosocial information gathered during pre-assessments, the patient’s capacity for self-management support and the health professional's applied knowledge of cancer-related fatigue management strategies. | 3b) Plan a culturally and contextually relevant fatigue self-management care plan drawing on clinical and psychosocial information gathered during pre-assessments, the patient’s capacity for self-management support **(including addressing identified barriers to self-management or leveraging specific strengths/ capabilities),** and the health professional's applied knowledge of cancer-related fatigue management strategies. |
| 3c) Incorporate the patient’s support networks (family, friends, carers) into action planning. | 3c) **Consider** **incorporating** the patient’s support networks (family, friends, carers) into action planning **with the patient’s consent.** |
| **DOMAIN 3: IMPROVING PATIENT KNOWLEDGE** | |
| **Key Practice 4** | |
| 4. Provide information (visual, written, verbal) on cancer-related fatigue and common management strategies | Provide **tailored evidence-based** information on cancer-related fatigue and common management strategies **in a diversity of formats to accommodate different learning styles.** |
| 4a) Inform the patient and their support network of the differences between cancer-related fatigue and ‘normal’ fatigue. | Reached consensus for inclusion in Round 1 and included in final framework |
| 4b) Inform the patient and their support network of the causes, key risk factors, presenting characteristics, and the possible effects and interferences of cancer-related fatigue on daily living. | Reached consensus for inclusion in Round 1 and included in final framework |
| 4c) Communicate tailored evidence-based information to the patient and their support network regarding the benefits of exercise and physical activity for managing cancer-related fatigue and have an awareness of the strength of such evidence. | Reached consensus for inclusion in Round 1 and included in final framework |
| 4d) Communicate tailored evidence-based information to the patient and their support network on other management strategies for cancer-related fatigue (along with their risk and benefits) and have an awareness of the strength of such evidence. | 4d) Communicate tailored evidence-based information to the patient and their support network on **psychological and complementary medicine** management strategies for cancer-related fatigue (along with their risk and benefits) and have an awareness of the strength of such evidence. |
| **Key Practice 5** | |
| 5. Provide tailored evidence-based information on managing common psychosocial consequences of cancer and cancer-related fatigue | Provide tailored evidence-based information on managing common psychosocial consequences of cancer and cancer-related fatigue **in a variety of formats to accommodate different learning styles.** |
| 5a) Provide the patient and their support network with evidence-based information on how psychological and psychosocial factors (e.g., fear of cancer recurrence or progression, anxiety, depression, and stress) contribute to, and are exacerbated by cancer-related fatigue. | Reached consensus for inclusion in Round 1 and included in final framework |
| 5b) Provide the patient with coping strategies for managing psychological and psychosocial factors | **This was deleted as panel feedback indicated it was already covered in Domain 4 (Key Practice 9).** |
| 5c) Provide the patient with coping strategies for processing and communicating with others about cancer-related fatigue and cancer experiences. | **This was deleted as panel feedback indicated it was already covered in Domain 4 (Key Practice 7).** |
| **Key Practice 6** | |
| 6. Provide information about available social support | **Provide tailored evidence-based information** about available social support **in a variety of formats to accommodate different learning styles and check patient understanding** |
| 6a) Facilitate the involvement of the patient’s support network (family members, friends, carers, significant others) in cancer-related fatigue self-management activities | 6a) **Consider** facilitating the involvement of the patient’s support network (family members, friends, carers, significant others) in cancer-related fatigue self-management activities **with the patient’s consent.** |
| 6b) Provide the patient and their support network with education and information about how to seek further social support and inform the patient of relevant support services in their community. | Reached consensus for inclusion in Round 1 and included in final framework |
| 6c) Facilitate the exchange of cancer and cancer-related fatigue experiences and/or discussions between the patient and other cancer survivors. | 6c) **Consider** facilitating the exchange of cancer and cancer-related fatigue experiences and/or discussions between the patient and other cancer survivors, **if the patient agrees.** |
| **DOMAIN 4: TRAINING REHEARSAL (STRATEGY BUILDING)** | |
| **Key Practice 7** | |
| 7. Provide the patient with problem solving and evidence-based solution-focused strategies to communicate with their systems of support (includes health professionals, non-health professionals, personal communities; and voluntary and community groups) about cancer-related fatigue. | Reached consensus for inclusion in Round 1 and included in final framework |
| 7a) Provide the patient with the skills to self-advocate and communicate with health professionals, non-health professionals, personal networks, employees, and others about cancer-related fatigue. | Reached consensus for inclusion in Round 1 and included in final framework |
| 7b) Provide coaching and counselling about navigating relationships and social support | Reached consensus for inclusion in Round 1 and included in final framework |
| **Key Practice 8** | |
| 8. Provide evidence-based coaching for lifestyle modifications that support living with cancer-related fatigue. | Reached consensus for inclusion in Round 1 and included in final framework |
| 8a) Provide the patient and their support network with evidence-based tailored coaching and practical strategies for exercise (e.g., aerobic, resistance, yoga, balance) and physical activity (e.g., daily walking, morning stretches). | Reached consensus for inclusion in Round 1 and included in final framework |
| 8b) Provide tailored coaching and practical strategies to the patient and their support network that supports the patient’s everyday activities | Reached consensus for inclusion in Round 1 and included in final framework |
| 8c) Refer the patient to relevant services and professionals for support when indicated (e.g., if you are unable to provide tailored coaching). | 8c) **Involve** and refer the patient to relevant services and professionals for support when indicated (e.g., if you are unable to provide tailored coaching). **and in the planning and decision making of the patient’s care.** |
| 8d) Involve relevant services and professionals in the planning and decision making of the patient’s care | **This was combined with practice component 8c) as per panel feedback (due to overlap).** |
| **Key Practice 9** | |
| 9. Provide the patient with evidence-based problem-solving strategies for coping with the psychological effects or risk factors of cancer-related fatigue. | Reached consensus for inclusion in Round 1 and included in final framework |
| 9a) Provide patients with strategies for coping with anxiety, fear of recurrence or progression, stress, depression, and managing interpersonal relationships. | Reached consensus for inclusion in Round 1 and included in final framework |
| **NEW** | 9b) Refer the patient to relevant services (e.g., psycho-social oncology) and professionals for support when they are unable to provide tailored coaching. (new) |
| **Key Practice 10** | |
| 10. Provide evidence-based general health promotion and education on lifestyle adaptation strategies. | Reached consensus for inclusion in Round 1 and included in final framework |
| 10a) Provide relevant general lifestyle advice and counselling support to the patient and their support network. | Reached consensus for inclusion in Round 1 and included in final framework |
| **DOMAIN 5: CARE CO-ORDINATION AND MAINTENANCE** | |
| **Key Practice 11** | |
| 11. Provide regular review of self-management goals and action plans in collaboration with the patient (and their social network). | Provide regular review of **self-management activities**, and self-management goals and action plans in collaboration with the patient, **their support network (with the patient’s consent), and their health care team.** |
| 11a) Reformulate previously established goals based off the patient's confidence, needs, and progress (i.e., goal attainment). | Reached consensus for inclusion in Round 1 and included in final framework |
| 11b) Establish long term goals prior to the conclusion of support to facilitate continual patient self-care beyond the self-management support program. | Reached consensus for inclusion in Round 1 and included in final framework |
| 11c) Provide scheduled reviews to monitor cancer-related fatigue (e.g., severity and lifestyle interference), associated symptoms (e.g., stress, depression), and review of progress with self-management behaviours (e.g., confidence, physical activity, activity management). | Reached consensus for inclusion in Round 1 and included in final framework |
| **Key Practice 12** | |
| 12. Provide practical support that facilitates ongoing self-management. | Reached consensus for inclusion in Round 1 and included in final framework |
| 12a) Provide practical support to assist the self-monitoring of cancer-related fatigue symptom reporting (e.g., fatigue severity, energy levels, and other factors that impacted fatigue), and behaviour change (e.g., exercise, mediation habits, physical activity, and dietary habits). | Reached consensus for inclusion in Round 1 and included in final framework |
| **Key Practice 13** | |
| 13. Be able available on request to review the symptoms of cancer-related fatigue. | Reached consensus for inclusion in Round 1 and included in final framework |
| 13a) Be available for symptom review, treatment modification or counselling if symptom thresholds are exceeded. | 13a) Be available for symptom review, treatment modification or counselling if symptom thresholds are exceeded **or if requested by the patient due to a change in support requirements.** |
| **Key Practice 14** | |
| Be able available on request to review the symptoms of cancer-related fatigue | **This was moved to a practice component of Key practice 13 (due to overlap) as per panel feedback** |
| 14a) Be available for symptom review, treatment modification and/ or counselling if symptom thresholds are exceeded | **This was merged with practice component 13a as per panel feedback.** |

**Online Resource 2:** Qualitative examples of identified themes

| **Themes** | **Theme frequency** | **Codes** | **Code frequency** | **Example quotes for theme** | **Response to theme** |
| --- | --- | --- | --- | --- | --- |
| **Round 1** | | | | | |
| Tailor information | 16 | Ensure cancer survivors and their family/caregivers understand the information provided | 6 | - Communication levels, jargon, making sure information is not just given but ensure understanding. - Important to check how information can be delivered since information can be tiring. Important also to address with how the patient and parents/network can use the information. “ - Establish an open rapport with patient rather than just providing written documentation. i.e., a video - Coping with information overload and having a support person to collate information - Provide the patient with..." Whenever I see this, I am reminded of how most of this information has just been given to us in a handout with little explanation or exploration. I would like to see a discussion aspect to those practices. The same with "Inform" - while that is the practice there needs to be a check for understanding. I have been informed of many things during medical appointments but never fully understood the implications. - It is important that they know their patients and tailor their messages to how the patient process this. Not everyone wants detailed information, so know your client! - Avoid jargon as most 'civilians' won't recognize or understand. People generally are embarrassed to ask the meaning. - Patients need information on their treatment & how to combat the side effects of both the disease & treatment, but only enough for their needs at that time. There is more than enough going on inside their head. - Medical professionals need to show empathy with the patient and family and not see the patient as a statistic and talk over them as if they are just a job. - Personalisation of provided information in partnership with the healthcare professional should also be mentioned. | An additional document was developed, emphasising the importance of underpinning the delivery of practices by the presence of effective, person-centred, health professional communication which involves the ability to establish and develop mutual understanding, rapport, trust, respect, and cooperation with people affected by cancer using clear and plain language.  Wording of practices in Domain 3 - (Improve Patient Knowledge) have been modified to reflect the need to provide information in a diversity of formats to accommodate different learning styles. |
|  |  | Find out patient's/ cancer survivors information style (e.g., how would they prefer information to be delivered). | 7 |  |  |
|  |  | Place emphasis on delivering information with care no to overload patient. | 3 |  |  |
| Consider patient preferences about involving support network | 14 | Consider patient preferences when involving support network in self-management support | 10 | - Consultation with a support network should be contingent on an individual's preference - Incorporation of support networks, if the patient so chooses / wishes. not every patient aims to exchange experiences with other patients (wording shood reflect this). - Must incorporate consent from patient into using the wider support network in action planning, not all patients want their families or friends involved. This should be worded as 'consider incorporating ... with patient's consent'. - Some patients are very shy of having their families/friends involved - they find it difficult to share how they feel. - Again, this can depend on the patient and how they feel about sharing - "Not all patients have the desire to include their support network into action planning so I think there should be more emphasis on the patient's needs" (Round 2) - Integration of evidence-based management strategies for managing fatigue, alongside consideration of patient priorities, cultural context, etc. - Patient consent to involvement of support network should be spelled out. | Wording changed to Key Practice 3 to acknowledge the need to ensure coping strategies are aligned with patient preferences.  Practices that involve the inclusion of support networks (peer, family/caregiver) have been adjusted to reflect the need to consider individual preference. |
|  |  | Consider patient preferences when involving other cancer survivors in self-management support | 2 |  |  |
|  |  | Consider patient preferences when collaboratively deciding the coping strategies to be incorporated in the patient's fatigue action plan | 2 |  |  |
| Consider the patient's support network | 4 | Consider what the patient's support network needs to assist the patient to self-manage. | 2 | - Identify caregivers/parents need of support from healthcare team for being able to support the patient - To what extent is fatigue degrading the patient's lifestyle and that of his partner/family? - Need to consider family. | A new practice component was added to Domain 1 (Establishing Context and Defining the Problem), Key Practice 1. |
|  |  | Consider how the patient’s cancer-related fatigue affects support network. | 2 |  |  |
| Focus on identifiable risk factors for fatigue | 10 | Focus on identifiable risk factors for fatigue | 10 | - Health professionals, specially doctors should also carry the efforts needed to exclude and treat aggravating causes to cancer fatigue, like anemia, hormonal disturbances, insomnia, pain,…. - Conduct testings that identify and eliminate other possibilities related to fatigue such as hormones, thyroid, fibromyalgia etc - Identify actionable risk factors for the fatigue itself. I practice in the malignant hematology setting, where we can often adjust or consider breaks of medications like lenalidomide or ibrutinib (being used for long-term disease control for MM or CLL, respectively) that can cause fatigue.” - Factors could be other disease factors or medical conditions and this should be assessed; and this should be addressed specifically. | Changes made to wording of practice component 1c) to explicitly specify that the purpose of information collection is to identify risk factors for cancer-related fatigue that can inform coping strategies. |
| Identify who should deliver practices and how | 10 | Specify constraints to executing practices | 4 | - This may need to be split up by [health professional] domain. Physicians like myself aren't equipped to do some of the tasks above, versus a social worker who might be able to provide direct coaching / counselling. - This comment pertains to all domains. All of this is great, but who, when and how will this care be delivered... - all the items are relevant. However, it is very much to do it all - very broad and should be focused on key precipitating or predisposing factors specific to fatigue; if we add all these expectations for managing fear of recurrence then fatigue support will not occur. perhaps engage other professional support for managing co-occurring depression, etc. but really focus on the key practice for fatigue. - At some point in the recommendations, there should be a reference on how different health care professionals, community services and everybody communicate and work as a care team. This concerns also the acquisition of competencies to work with this population as in the exercise field, psychology, nutrition, social,.... - assume ahead of time that the health professional (clinician or nurse?) will have the capacity to deliver these practice components. Is there not a need to specify such constraints - or an absence of them? - It should be an MDT (multidisciplinary team) model of working involving the team's expertise (with referrals if/as necessary) | Referral and involvement of multidisciplinary healthcare team is already incorporated in Practice Component 8b. A new practice component specifically highlighting referral for managing psychological effects (Practice Component 9b) has been created and put forward for rating and feedback in Round 2.  An additional document providing detail on the execution of the key practices and practice components (i.e., who and how) has been created and added to the second-round survey for participants to peruse. Acknowledges that cancer-related fatigue is multi-factorial and thus will require a multidisciplinary health team to manage.  This study presents best practices, implementation of the framework (including how tasks and responsibilities can be shared amongst the healthcare team will be investigated in next research phase. |
|  |  | Specify the multidisciplinary nature of self-management support and the need to engage with other professional support | 3 |  |  |
|  |  | Specify what health profession does what. | 3 |  |  |
| Positive attitudes to framework | 10 | Framework is a useful and comprehensive tool. | 3 | - Just a comment that this is a very comprehensive set of practices that should, in fact, be in place for every survivor not just those with fatigue. It is so relevant to the many issues we face once treatment has ended and there is no path for us to follow. Most of our family doctors are not aware of the complications we face, and the cancer centres are focussed on active treatment, so we tend to feel abandoned. Thank you for the opportunity to be part of a solution. I would like to think that these practices would become part of our normal follow-up. I look forward to the next stage. - [About facilitating exchange between cancer survivors] To me this is by far the most important. Everything I have learnt in cancer management is from other cancer survivors belonging to the same demographics as me, worldwide in 6 different languages, all on social media. And NO it is NOT Facebook nor Dr Google, we are organised in subreddit or twitter discussion groups, all have PhDs, access to research publications, used to do scoping, subscribe to up to date, follow short specialist courses on line all over the world etc ... we are just ill and do not put Dr in front our name . We then disseminate the knowledge through the closed Facebook groups so patients can drive their care more effectively and co-design, co-create instead of being ""managed "". The idea is to learn how to address our own needs with the support of professionals rather than professionals trying to support our needs, total shift in approach. - [About facilitating exchange between cancer survivors] It helps to speak to someone who understands - The specific strategies and examples provided are useful - These all seem like good ideas! - Employers should also be aware of the plan to the extent of documented support. Often an employer feels that because an employee 'looks' ok that he/she can do the same job as always. They do not see the fatigue after work, the length of preparation it takes to get ready for work, or the fatigue during the day. As a patient, we tend to hide our illness from employers from fear of being let go or transferred to a less appealing job. - This is a very important symptom of cancer treatment and should be given more importance in follow up (Round 2) - The patient should be given a plan as its difficult to self-motivate when you are feeling so low and tired (Round 2) | We thank the panel for their feedback. |
|  |  | Incorporation of exchange between cancer survivors and support network is important. Glad this component is included in the framework | 2 |  |  |
|  |  | Examples and strategies provided are useful for framework application. | 4 |  |  |
|  |  | Glad support with communicating fatigue to employers has been included in the framework | 1 |  |  |
| Focus on referrals | 6 | Engage community services in provision of support. | 6 | - Holistic practical approach to managing fatigue not just limited to health environment but other pathways to access assistance where financially strapped to access medications gym yoga physio therapies etc - Connect patient with community services so people with no one in the world are not left to die alone and receive some help. - Helpful to inform individuals about available community-based resources for fatigue management - Include appropriate referrals to other agencies and professionals with patient engagement and consent. | It is agreed that community engagement is essential. The involvement of community services has already been incorporated (particularly Domain 2 – developing an action plan - in practice component 6b.) Referral and involvement of multidisciplinary healthcare team already incorporated in Practice 8b. A new practice component specifically highlighting the need for providing referrals when managing psychological effects (9b) was created. |
|  |  | Engage and refer to other health professions. | 2 |  |  |
| Assist implementation of framework | 6 | Include more examples to aid implementation of framework practices | 4 | - Recommend clearer linkage to cancer-related fatigue, as currently it is quite broad. Adding examples of strategies can also aid in implementation. - Could automate [the collection of clinical, behavioural, and symptom information] with questionnaires the way that InA (from Savor Health - a text-based oncology nutrition artificial intelligence does): https://www.savorhealth.com/ - [About provision of fatigue information] A patient brochure would be extremely helpful! - Are the action plans co-created with patients in words or all in infographics? multiple choice? - Strategies provided are very useful, recommend including for all practices. | An accompanying document providing further information on the framework and context was created. All strategies and further examples of items in practice framework were moved to this document. Document will be distributed to panel participants in Round 2 for their perusal. |
|  |  | Consider using tools to assist with creating action plans and collecting information | 2 |  |  |
| Identify the applicability of framework | 2 | Consider the relevance of framework to underserved populations | 2 | - [Self-management support is] something for the privileged: people who have time to be concerned about their individual wellness rather than the collective, middle class affluent people. Obviously not directed to refugees. We too have cancer. - other pathways to access assistance where financially strapped to access medications gym yoga physio therapies etc | We agree that further targeted consultation with specific stakeholders is needed to understand relevance and applicability for different groups. Cultural influences on health and disease, and attitudes towards care will need to be considered when adapting the framework to different contexts in the next step. |
| Round 2 | | | | | |
| Provide fatigue specific strategies and examples | 7 | Contextual elements missing | 7 | - Contextual element missing? (Home, work, clubs, community organisations)" - Incorporate fatigue specific self-management strategies and strategies for coping with fatigue. - Coping strategies for management of fatigue is relevant but this should also include fatigue-specific self-management strategies (i.e., adaptive pacing, physical activity, etc). So word as incorporates fatigue specific self-management strategies and strategies for coping with fatigue. - While tailoring is ideal, what is missing is clarity regarding the characteristics on which the provision of information should be tailored (i.e., symptom severity? resources available? intervention preferences?).” - Greater specificity would be helpful if these practice components would be used for teaching or evaluating practice. Perhaps "attend to requests for further symptom review, treatment modification, or counselling..." - I have a problem with adjective "" general "" i would like it to be targeted and " contextually relevant" otherwise, waste of time. We are already bombarded by these messages constantly so totally disregard it. | Strategies and examples of practice scenarios were included in a separate document attached to the survey. Further analysis shows not all panel participants opened document. Panel feedback shows inclusion of these strategies and examples are essential. They have now been presented clearly. |
