# Supplementary material for: Development of a self-management support practice framework for addressing cancer-related fatigue: a modified Delphi study
Source: J Cancer Surviv. 2023 Feb 24;18(3):972–82. doi: 10.1007/s11764-023-01348-7 (PMC11082027; doi:10.1007/s11764-023-01348-7)
Supplement: Supplementary file 2 — Supplementary file2 (PDF 397 KB) [file 11764_2023_1348_MOESM2_ESM.pdf]

CANCER-RELATED FATIGUE SELF-MANAGEMENT SUPPORT PRACTICE FRAMEWORK

CONTEXT

Cancer-related fatigue is one of the most common and distressing symptoms reported by people affected by cancer [1]. There is need for specific guidance [2, 3] to assist health professionals in facilitating the management of cancer-related fatigue [4, 5].

This Practice Framework presents the core practices required by health professionals to deliver effective self-management support to cancer survivors (pre- and post-cancer treatment) experiencing cancer-related fatigue, and presents an outline that is pragmatic, contextually adaptable, and clinically relevant.

The practices included in this framework revolve around an individual's perceived level of fatigue severity and interference, regardless of cancer diagnosis, treatment type, or phase in the cancer care continuum. This framework presents the core tasks that should be undertaken when providing support for cancer-related fatigue management, rather than explore changes within or between any subgroups.

COMPONENTS OF THE CANCER-RELATED FATIGUE SELF-MANAGEMENT SUPPORT PRACTICE FRAMEWORK

DOMAINS

The practices included in this framework have been categorised into five domains. The domains of the practice framework provide a broad thematic organization for the self-management support practices and strategies. Each domain addresses a different set of essential processes that should be undertaken to sufficiently support cancer survivors to manage their cancer-related fatigue.

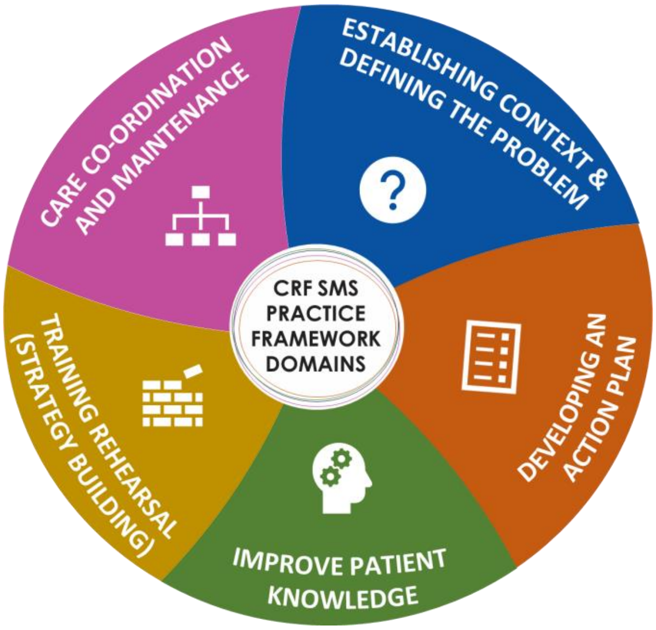

Each domain consists of corresponding Key Practices and Practice Components. The content of these Key Practices and Practice Components have been informed by:

(i) a systematic review of self-management support programs for cancer-related fatigue,

(ii) The Capabilities for Supporting Prevention and Chronic Condition Self-Management Framework

KEY PRACTICES

Key practices describe the proposed activities health professionals are required to undertake to provide best practice self-management support for cancer-related fatigue.

PRACTICE COMPONENTS

Practice components describe the steps needed to complete a key practice. Health professionals are expected to demonstrate all practice components for all the key practices when providing self-management support for cancer-related fatigue. These practice components may require health professionals to apply, adapt, and integrate new and existing evidence-based knowledge.

HOW CAN THIS FRAMEWORK BE USED?

Cancer-related fatigue is multifactorial – meaning that there are a diverse range of factors that can contribute to, or cause it (e.g., cancer type, cancer treatment type, anaemia, nutrition factors, psychological factors, etc.); therefore, it is expected that the **provision of management support will require a multi-disciplinary approach**. It is not expected that all health professionals be proficient in all practice components, whereby certain practices may fall outside of one's professional expertise or scope (e.g., radiation therapist providing psychological coping strategies). The provision of support for cancer-related fatigue management requires professional collaboration between differing specialities within a multidisciplinary care approach which includes an informed and supportive health care team.

The execution of the key practices and practice components specified in this document may require health professionals to apply, adapt, and integrate new and existing evidence-based knowledge. This practice does not provide, present, or describe the capabilities or competencies required by health professionals. There are already clinical practice guidelines outlining evidence-based management strategies for cancer-related fatigue (e.g., NCCN, CAPO, Macmillan, other (Level I) empirical evidence). Instead, this practice framework outlines the tasks that health professionals and or health care teams should undertake when supporting people affected by cancer to manage their fatigue.

Finally, the execution of practices outlined in the framework should be underpinned by the presence of effective, person-centred, health professional communication which involves the ability to establish and develop mutual understanding, rapport, trust, respect, and cooperation with people affected by cancer using clear and plain language. This includes making appropriate adjustments (e.g., use of appropriate language and detail, use of appropriate verbal and non-verbal cues, confirming that the other person has understood) to meet the communication and information needs of patients and their support network (e.g., caregivers, family, friends) and providing opportunities for the patient and their support network to demonstrate their understanding.

Use of the Practice Framework

The following recommendations are designed to help individuals and organisations make the best possible use of the Practice Framework.

For the individual health professional

- Use the Practice Framework as a tool:
  - to guide the provision of self-management support for cancer-related fatigue
  - for determining your professional development needs
  - for evaluating current practice when providing support to those managing cancer-related fatigue
- Use the Practice Framework and associated learning resources to undertake self-directed learning.
- Refer colleagues new to working with people affected by cancer (i.e., cancer survivors, cancer patients) and/or cancer-related fatigue to the framework.
- Use the Practice Framework to develop an understanding about:
  - the extent of cancer-related fatigue impact on those affected by cancer and the importance of its management
  - the various roles of different health professionals in the delivery of self-management support for cancer-related fatigue

For the clinical leader/ educator

- Use the Practice Framework as a tool to:
  - develop clinician awareness and knowledge of evidence-based cancer-related management and assessment strategies
  - provide training/in-service programs to improve ability to undertake practices (how to use certain questionnaires, practice developing an action plan, etc.)
  - advocate for system-level changes to provide resources (time, space, and human) to deliver optimal support for cancer-related fatigue management

For the cancer-related fatigue self-management intervention/program developer

- Use the Practice Framework to aid development of a cancer-related fatigue self-management intervention/program (determining the specific components that are needed).

For the researcher

- Use the Practice Framework as a tool to:
  - describe self-management support interventions for cancer-related fatigue
  - synthesize evidence on cancer-related fatigue self-management

For the consumer experiencing cancer-related fatigue (and their family/carer's)

- Use the Practice Framework as a tool to:
  - develop understanding of the various roles of different health professionals in the delivery of self-management support for cancer-related fatigue
  - Advocate for improved delivery of cancer-related fatigue management support.

advise your health care team(s) about the existence of the practice framework and teaching and learning resources in efforts to improve your care.

CANCER-RELATED FATIGUE SELF-MANAGEMENT SUPPORT PRACTICE FRAMEWORK

***Note 1**: The examples 'tools, strategies, and prompts' section provides a guide to help health professionals consider the support expected. These descriptions provide examples but are not intended to be exhaustive. Information for the 'tools, strategies, and prompts' section was derived from the NCCN Cancer-related Fatigue Guidelines [6] and a systematic review of self-management support programs for cancer-related fatigue [7].
****Note 2:** The 'patient' refers to the individual experiencing cancer-related fatigue (encompasses those who are on active cancer treatment and those who have completed active cancer treatment).

Domain 1: Establishing Context and Defining the Problem

Key Practice 1	Practice Components	*Examples of Tools, Strategies, Prompts, etc.
1. Collect and use clinical and behavioural information to inform decision making about the patient's self-management of cancer-related fatigue.	1a) Conduct a consultation and assessment with the **patient, and if available, other key people in the patient's support network (with the patient's consent), to collaboratively define key concerns, problem areas, and priorities.	**Things for clinicians to consider**<br>• How is fatigue impacting the patient's daily activities (social life, work life, personal feelings)? Examples of cancer-related fatigue presentation in patients can include lack of energy; lack of concentration; inability to complete daily tasks such as housework; poor concentration [6].<br>• Consider involving the patient's support network in information collection [6, 7]. Oftentimes the patient may not be aware that fatigue has negatively affected their life: however, their support network may be more cognizant of changes and the effect of fatigue [6].
	1b) Identify family members/informal caregivers need for guidance from healthcare providers to facilitate their ability to support the patient with self-management strategies.	
	1c) Collect and record key clinical, behavioral, and psychological information and highlight clinical, behavioural or psychological risk factors that may contribute to the patient's cancer-related fatigue.	**<u>Clinical information can include:</u>** cancer treatment type, cancer treatment length, cancer recurrence or progression, referral history, age, medication history, pain levels [6]<br>**<u>Clinical risk factors of cancer-related fatigue can include:</u>** anaemia, chemoradiation or other systemic treatments, hypothyroidism, hormonal cancer treatment depression, anxiety, stress, fluid/ electrolyte imbalance, pain<br>**<u>Behavioural information can include:</u>** sleep habits, current level of physical activity, diet, confidence in engaging in self-management behaviours<br>**<u>Behavioural risk factors of cancer-related fatigue can include:</u>** sleep disturbances, inactivity, poor diet<br>**<u>Psychological information can include:</u>** psychiatric history, history of depression and anxiety<br>**<u>Psychological risk factors of cancer-related fatigue can include:</u>** depression, anxiety, stress
	1d) Conduct a fatigue assessment to determine fatigue severity, onset, duration, pattern, associated patient distress, and interference with daily living.	**Things for clinicians to consider [6]**<br>• Brief, self-reported, quantitative, and single-item assessments with empirically established cut-off scores are effective.<br>• E.g., 0 to 10 numerical rating scale (0 = no fatigue and 10 = worst fatigue), mild fatigue indicated as a score of 1 to 3, moderate fatigue as 4 to 6, and severe fatigue as 7 to 10.

		• Evaluation of fatigue in children can be modified to a scale of 1 to 5 or asked if they are 'tired' or 'not tired'.

Key Practice 2	Practice Component	Example of Tools, Strategies, Prompts, etc.
2. Assess the patient's capacity for self-management.	2a) Identify factors within the patient, and the patient's physical and social environment, that they perceive may improve or worsen their cancer-related fatigue.	**Things for clinicians to consider [7]**.<br>Are there things the patient and/or the patient's support network have noticed that makes their fatigue worse or better? (For example, patients can describe worse fatigue on treatment days; more severe fatigue in different parts of the day; changes during certain weather)
	2b) Identify the patient's (and their informal caregiver's/ support network's) beliefs, attitudes, and knowledge about cancer-related fatigue, including differences between patients and informal caregivers, and identify their current coping strategies.	**Things for clinicians to consider [7]**<br>• What does the patient and their support network know about cancer-related fatigue?<br>• How has the patient managed (or been managing) their fatigue? (e.g., What strategies have they tried, and not found success with? What do they feel has worked?)
	2c) Identify factors (i.e., cultural consideration, language literacy levels, availability of peer support network, pre-existing conditions) that may affect the patient's ability to participate in self-management activities.	**Things for clinicians to consider [6, 7]**<br>• What factors might affect the patient's ability to undertake exercise and other fatigue management activities (e.g., lymphoedema, neuropathy in fingers, lack of transport, financial status, physical status, lack of support to assist with daily tasks.)<br>• How does the patient's informal caregivers/ support network feel about providing assistance with support?

Domain 2: Developing an Action Plan

Key Practice 3	Practice Component	Example of Tools, Strategies, Prompts, etc.
3. Create a cancer-related fatigue management action plan* in collaboration with the patient that incorporates evidence-based coping strategies that are aligned with patient preferences.	3a) Reflect on the patient's capacity for self-management (including barriers), and the clinical and behavioural information gathered during pre-assessments, to refine the patient's priorities, needs, and goals concerning their cancer-related fatigue and general lifestyle.	**Things for clinicians to consider [6, 7]**<br>• What does the patient wish to get out of the support provided?<br>• How motivated is the patient to address their cancer-related fatigue?<br>• A particular patient may need more support network engagement (health professionals, friends, family, carer) and structural involvement versus another who may be highly self-motivated.<br>• Consider using a readiness ruler to guide conversations around priorities, importance, confidence, and personal change.
	3b) Plan a culturally and contextually relevant fatigue self-management care plan. The plan should be informed by clinical and psychosocial information gathered during pre-assessments, the patient's capacity for self-management (including addressing identified barriers to self-management or leveraging specific strengths/ capabilities), and the health professional's applied knowledge of cancer-related fatigue management strategies.	**Culturally and contextually relevant** takes into consideration socio-cultural factors (e.g., cultural and linguistic diversity, age, gender, disability, religion, socioeconomics, geographical locations) and acknowledges and incorporates the importance of culture, the assessment of cross-cultural relations, and the adaptation of services to meet culturally unique needs.<br><br>**Things for clinicians to consider [7]**<br>• Ensure goals are SMART (specific, measurable, actionable, realistic, time-bound), patient-centered, and include specific actions for moderately challenging behaviors.<br>• Goal development process may need to include motivational interviewing techniques to address patient priorities and concerns; rolling with resistance when required, and facilitating the patient's self-agency
	3c) Consider incorporating the patient's support networks (family, friends, carers) into action planning with the patient's consent.	**Things for clinicians to consider**<br>• The inclusion of a support network in action planning can increase uptake of fatigue self-management strategies [7].<br>• How can the patient's support network assist the patient to achieve goals in action plan?<br>• For example, in a situation of escalating fatigue at the end of life, family members may wish to designate individuals to assume activities relinquished by the individual with cancer [6].

***Definition/ components of a Cancer-related fatigue Action Plan**: The fatigue self-management action plan should be developed in collaboration with the patient and (if available) their other supports (including carers). The care plan should facilitate the patient's engagement in their own care (leveraging the patient's strengths and capabilities) and contain patient defined problems and goals, community education programs and resources, community support networks, allocated time for review and follow-up.

Domain 3: Improving Patient Knowledge

<table>
<tr><th>Key Practice 4</th><th>Practice Component</th><th>Example of Tools, Strategies, Prompts, etc.</th></tr>
<tr><td rowspan="4">4. Provide tailored evidence-based information on cancer-related fatigue and common management strategies in a diversity of formats to accommodate different learning styles</td><td>4a) Inform the patient and their support network of the differences between cancer-related fatigue and ‘normal’ fatigue.</td><td>Cancer-related fatigue vs. normal fatigue: Cancer-related fatigue is a physical, emotional and/or cognitive tiredness that is more persistent and severe than normal fatigue and cannot be relieved by adequate sleep and rest [6].
Things for clinicians to consider [6]
• There is a need to relay to the patient that if fatigue does occur often, it is not necessarily an indication that the treatment is not working or that the disease is progressing/recurring.
• Reassurance is important as fear of progression is a main reason for the under-reporting of fatigue.</td></tr>
<tr><td>4b) Inform the patient and their support network of the causes, key risk factors, presenting characteristics, and the possible effects and interferences of cancer-related fatigue on daily living.</td><td>Examples of risk factors for cancer-related fatigue [6]
Insomnia; depression, anxiety and stress, pain; being overweight, anaemia; sudden weight loss, poor diet, other medical conditions (e.g., heart problems, diabetes).
Examples of presenting characteristics and interference on daily living [6]
Difficulty making decisions, sleeping, maintaining attention and concentration; poor memory, feeling drained with no energy or strength, inactivity, low mood.</td></tr>
<tr><td>4c) Communicate tailored evidence-based information to the patient and their support network regarding the benefits of exercise and physical activity for managing cancer-related fatigue and have an awareness of the strength of such evidence.</td><td>• Several meta-analyses have been conducted to provide a comprehensive evaluation of the impact of increased activity upon cancer-related fatigue. Studies suggest that exercise and physical activity has a beneficial effect on fatigue in patients during and after cancer treatment [6].
• It is reasonable to encourage patients to engage in a moderate level of physical activity (3 to 5 hours per week) during and after cancer treatment [6].</td></tr>
<tr><td>4d) Communicate tailored evidence-based information to the patient and their support network on psychological and complementary medicine management strategies for cancer-related fatigue (along with their risk and benefits) and have an awareness of the strength of such evidence.</td><td>Examples of management strategies (excluding physical activity and exercise) [6]
Energy conservation, sleep therapy, activity management, counselling, cognitive behavioural therapy.</td></tr>
</table>

Key Practice 5	Practice Component	Example of Tools, Strategies, Prompts, etc.
5. Provide tailored evidence-based information on managing common psychological consequences of cancer and cancer-related fatigue in a variety of formats to accommodate different learning styles	5a) Provide the patient and their support network with evidence-based information on how psychological and psychosocial factors (e.g., fear of cancer recurrence or progression, anxiety, depression, and stress) contribute to, and may be exacerbated by cancer-related fatigue.	**Things for clinicians to consider [6]**<br>• There is a strong correlation between emotional distress (depression, anxiety), stress, and cancer-related fatigue.<br>• Psychological and symptom distress also have a negative effect on the patient's confidence to undertake fatigue self-management behaviours.

Key Practice 6	Practice Component	Example of Tools, Strategies, Prompts, etc.
6. Provide tailored evidence-based information about available social support in a variety of formats to accommodate different learning styles and check patient understanding	6a) Consider facilitating the involvement of the patient's support network (family members, friends, carers, significant others) in cancer-related fatigue self-management activities with the patient's consent.	**Things for clinicians to consider**<br>• The availability of dependable caregivers can significantly impact the functional, emotional, and financial capacity of a patient and impact their pursuant fatigue [6].<br>• Involving the support network in self-management solutions (i.e. family members, exercising with the patient) may increase compliance/adherence to cancer-related fatigue self-management strategies [7].<br>• A support network can be particularly key when a patient lacks the economic and supportive resources to obtain tangible support [6].
	6b) Provide the patient and their support network with education and information about how to seek further social support and inform the patient of relevant support services in their community.	**Things for clinicians to consider**<br>• What are the available community-based resources for fatigue management?<br>• Are there community options for physical activity programs?<br>• What are the offerings of local advocacy groups?
	6c) Consider facilitating the exchange of cancer and cancer-related fatigue experiences and/or discussions between the patient and other cancer survivors (peer support), if the patient wishes to do so.	**Practical support strategies [7]**<br>Exchange can occur through in-person discussion groups, patient stories (written), patient videos, online forums.<br>**Things for clinicians to consider [6]**<br>Use a guided group setting for peer exchange to avoid well-meaning but non-evidence-based suggestions and to allow for consideration of whether someone's experience is truly relevant to another person. Also take into consideration groups being limited to the right stage (in-treatment for primary cancer, living with secondary cancer, etc.).

Domain 4: Training Rehearsal (Strategy Building)

Key Practice 7	Practice Component	Example of Tools, Strategies, Prompts, etc.
7. Provide the patient with problem solving and evidence-based solution-focused strategies to communicate with their systems of support (includes health professionals, non-health professionals, personal communities; and voluntary and community groups) about cancer-related fatigue.	7a) Provide the patient with the skills to self-advocate and communicate with health professionals, non-health professionals, personal networks, employees, and others about cancer-related fatigue.	**Strategies can include [7]**<br>• Providing examples of questions to ask healthcare professionals and examples of goals to prepare for discussions (e.g., “Tomorrow morning I will write a list of questions about fatigue to take with me to my appointment with the nurse next week.”)<br>• Advising patients to show their fatigue diary to their healthcare teams, and invite their significant others to accompany them to consults and explain fatigue interference in terms of everyday life (e.g., feeling drained, having difficulty climbing stairs, cooking etc.)<br>• Providing guidance on how to ask clarifying question**s.**
	7b) Provide coaching and counselling about navigating relationships and social support.	

Key Practice 8	Practice Component	Example of Tools, Strategies, Prompts, etc.
8. Provide evidence-based coaching for lifestyle modifications that support living with cancer-related fatigue.	8a) Provide the patient and their support network with evidence-based tailored coaching and practical strategies for exercise (e.g., aerobic, resistance, yoga, balance) and physical activity (e.g., daily walking, morning stretches).	**Things for clinicians to consider [6]**<br>Improved satisfaction with physical activity is often reported when family involvement in a program increases.<br>Encourage patients to engage in a moderate level of physical activity (at least 30 minutes a day <u>OR</u> at least 3 to 5 hours per week) during and after cancer treatment.<br>It is critical that:<br>• Patients choose a type of exercise they enjoy (as this increases the likelihood that they engage in physical activity)<br>• Providers discuss specific implementation strategies (type of exercise, time of day, days of the week, location of activity) to enable patients to make frequent activity a reality.<br>• Activity prescribed is individualized based on age, gender, cancer type, and fitness level.<br>• Activity begins with a low level of intensity and duration and is modified as the patient’s condition changes.
	8b) Provide tailored coaching and practical strategies to the patient and their support network that supports the patient’s everyday activities.	**Strategies can include** coaching on meditation, relaxation, and breathing exercises; providing nutritional consultation; coaching on changing sleep habits; providing advice on re-structuring daily activities and energy conservation; cognitive behavioural therapy [7].<br>**Examples of energy conservation strategies [6]**<br>• Maintaining a fatigue diary which will allows the patient to ascertain peak energy periods, and then plan their activities accordingly within a structured routine.<br>• Labor-saving techniques (wearing bathrobe instead of drying off, assistive devices such as walker, etc.)

Key Practice 8	Practice Component	Example of Tools, Strategies, Prompts, etc.
		**Examples of sleep hygiene strategies [6]**<br>• Going to bed and waking up at the same time each night, getting out of bed after 20 minutes if unable to fall asleep, avoiding long or late afternoon naps, avoiding caffeine in the afternoon.
	8c) Refer the patient to relevant services and professionals for support when indicated, and involve these services in the planning and decision making of the patient's care.	**Examples of relevant services or health professions**<br>• Referral to exercise specialists (e.g., physical therapist, physical medicine, exercise physiologist, rehabilitation specialist) for an assessment and provision of exercise prescription.<br>• Referral to cancer specific exercise programs<br>• Community outreach programs

Key Practice 9	Practice Component	Example of Tools, Strategies, Prompts, etc.
9. Provide the patient with evidence-based problem-solving strategies for coping with the psychological effects or risk factors of cancer-related fatigue.	9a) Provide patients with strategies for coping with anxiety, fear of recurrence or progression, stress, depression, and managing interpersonal relationships.	**Can occur through [7]:** Coaching on interpersonal relationships, distress management, sharing experiences with family and other cancer survivors, and assignments for building mental and social activity.<br>**Specific strategies can include [7]:**<br>• Cognitive restructuring and reframing; relaxation exercises; stress-management, breathing exercises, counselling, mindfulness therapy.<br>• Activating positive emotions, overcoming depressive periods, breathing exercises, mindfulness therapy.
	9b) Refer the patient to relevant services (e.g., psycho-social oncology) and professionals for support when you are unable to provide tailored coaching.	**Examples of relevant services or health professions**<br>Psychologist, behavioural therapist, counsellor, social worker, support network, community organisations.

Key Practice 10	Practice Component	Example of Tools, Strategies, Prompts, etc.
10. Provide evidence-based health promotion and education on lifestyle adaptation strategies.	10a) Provide relevant lifestyle advice and counselling support to the patient and their support network.	**Examples of relevant lifestyle advice**<br>Promotion and support for physical activity, exercise, sleep hygiene, diet, activity management and regulation, stress management, and general health and wellbeing (e.g., preventing weight gain, health nutrition, increasing fluid intake, anger management).

Domain 5: Care Co-ordination and Maintenance

Key Practice 12	Practice Component	Example of Tools, Strategies, Prompts, etc.
12. Provide practical support that facilitates ongoing self-management.	12a) Provide practical support to assist the self-monitoring of cancer-related fatigue symptom reporting (e.g., fatigue severity, energy levels, and other factors that impacted fatigue), and behaviour change (e.g., exercise, mediation habits, physical activity, and dietary habits).	**Support Strategies can include [7]**<br>Providing diaries, logs, activity trackers, pedometers, heart rate monitors, frequent telephone calls, automated email/text message reminders, home visits.<br>**Things for clinicians to consider [7]**<br>• Consider providing equipment that enables the patient to engage in physical activity or exercise in their homes.<br>• Consider recording self-management support coaching/consult sessions to facilitate home practice.

Key Practice 11	Practice Component	Example of Tools, Strategies, Prompts, etc.
11. Provide regular review of self-management activities, and self-management goals and action plans in collaboration with the patient, their support network (with the patient's consent), and their health care team	11a) Reformulate previously established goals based off the patient's confidence, needs, and progress (i.e., goal attainment).	**Things for clinicians to consider [7]**<br>• Review should incorporate constant encouragement to improve fatigue self-management engagement and confidence.
	11b) Establish long-term goals prior to the conclusion of support to facilitate continual patient self-care beyond the self-management support program.	
	11c) Provide scheduled reviews to monitor cancer-related fatigue (e.g., severity and lifestyle interference), associated symptoms (e.g., stress, depression), and review of progress with self-management behaviours (e.g., confidence, physical activity, activity management).	**Things for clinicians to consider [7]**<br>• Scheduled reviews can occur during consultations or logbook/diary review.

Key Practice 13	Practice Component	Example of Tools, Strategies, Prompts, etc.
13. Attend to requests to review the symptoms of cancer-related fatigue.	13a) Attend to requests (from the patient) for further symptom review, treatment modification or counselling if symptom thresholds are exceeded or if requested by the patient due to a change in support requirements.	**Examples include** facilitating additional counselling on request; ensuring patient accessibility to care coordinators, nurse practitioners, and psychologists for additional support.

References

1. Weis J, Horneber M. Cancer-Related Fatigue. Tarporley, United Kingdom: Springer Healthcare Iberica; 2014.

2. Pearson E, Morris M, McKinstry C. Cancer related fatigue: Implementing guidelines for optimal management. BMC Health Services Research. 2017;17.

3. Ann MB, Kathi M. Dissemination and Implementation of Guidelines for Cancer-Related Fatigue. Journal of the National Comprehensive Cancer Network J Natl Compr Canc Netw. 2016;14(11):1336-8.

4. Hilarius DL, Kloeg PH, van der Wall E, Komen M, Gundy CM, Aaronson NK. Cancer-related fatigue: clinical practice versus practice guidelines. Supportive Care in Cancer. 2011;19(4):531-8.

5. Jones G, Gollish M, Trudel G, Rutkowski N, Brunet J, Lebel S. A perfect storm and patient-provider breakdown in communication: two mechanisms underlying practice gaps in cancer-related fatigue guidelines implementation. Supportive Care in Cancer. 2021;29(4):1873-81.

6. Berger. NCCN clinical practice guidelines in oncology (NCCN Guidelines®) cancer-related fatigue. 2019.

7. Agbejule OA, Hart NH, Ekberg S, Crichton M, Chan RJ. Self-management support for cancer-related fatigue: A systematic review. International Journal of Nursing Studies. 2022;129:104206.
